# Chromosomal evolution among leaf-nosed nectarivorous bats – evidence from cross-species chromosome painting (Phyllostomidae, Chiroptera)

**DOI:** 10.1186/1471-2148-13-276

**Published:** 2013-12-26

**Authors:** Cibele G Sotero-Caio, Marianne Volleth, Lauren S Gollahon, Beiyuan Fu, William Cheng, Bee L Ng, Fengtang Yang, Robert J Baker

**Affiliations:** 1 Department of Biological Sciences, Texas Tech University, Lubbock, TX 79409, USA; 2 Department of Human Genetics, Otto-von-Guericke University, Magdeburg, Germany; 3 The Wellcome Trust Sanger Institute, Wellcome Trust Genome Campus, Cambridge, UK

**Keywords:** Karyotype evolution, Glossophaginae, Lonchophyllinae, *Macrotus*, Zoo-FISH

## Abstract

**Background:**

New World leaf-nosed bats, Phyllostomidae, represent a lineage of Chiroptera marked by unprecedented morphological/ecological diversity and extensive intergeneric chromosomal reorganization. There are still disagreements regarding their systematic relationships due to morphological convergence among some groups. Their history of karyotypic evolution also remains to be documented.

**Results:**

To better understand the evolutionary relationships within Phyllostomidae, we developed chromosome paints from the bat species *Macrotus californicus*. We tested the potential of these paints as phylogenetic tools by looking for chromosomal signatures in two lineages of nectarivorous phyllostomids whose independent origins have been statistically supported by molecular phylogenies. By examining the chromosomal homologies defined by chromosome painting among two representatives of the subfamily Glossophaginae (*Glossophaga soricina* and *Anoura cultrata*) and one species from the subfamily Lonchophyllinae (*Lonchophylla concava*), we found chromosomal correspondence in regions not previously detected by other comparative cytogenetic techniques. We proposed the corresponding human chromosomal segments for chromosomes of the investigated species and found two syntenic associations shared by *G. soricina* and *A. cultrata*.

**Conclusion:**

Comparative painting with whole chromosome-specific paints of *M. californicus* demonstrates an extensive chromosomal reorganization within the two lineages of nectarivorous phyllostomids, with a large number of chromosomes shared between *M. californicus* and *G. soricina*. We show that the evolution of nectar-feeding bats occurs mainly by reshuffling of chiropteran Evolutionarily Conserved Units (ECUs). Robertsonian fusions/fissions and inversions seem to be important modifiers of phyllostomid karyotypes, and autapomorphic character states are common within species. *Macrotus californicus* chromosome paints will be a valuable tool for documenting the pattern of karyotypic evolution within Phyllostomidae radiation.

## Background

New World leaf-nosed bats, family Phyllostomidae, represent the outcome of a remarkable burst of adaptive radiation and are the most diverse monophyletic group in terms of dietary specializations within higher vertebrates [1]. Besides their extensive morphological adaptations to distinct feeding strategies, evolution of Phyllostomidae has also been characterized by intense chromosomal reorganization in different lineages [2]. Although several studies have attempted to elucidate the chromosomal evolution in the group, especially during the early 80’s when major efforts were made towards the understanding of their intergeneric chromosome homologies and variability, karyotypic studies of the family have been hindered by two major factors: 1) absence of documented homology of G-bands to a common evolutionary origin that could potentially result in the grouping of non-related taxa as members of monophyletic units and; 2) absence of a statistically supported phylogenetic tree documenting intergeneric relationships to understand the order and place of events that shaped the karyotypes of current species [2,3].

Recent improvements in both molecular and cytogenetic techniques have addressed some of these difficulties. Of these, chromosome painting is the method of choice for reconstructing ancestral chromosomal associations in comparative interspecific research. This is based on the fact that homology is established mainly on the basis of DNA content, rather than banding similarity alone [4-8]. Additionally, the recent molecular phylogenies based on DNA sequence data of both mitochondrial and nuclear genes have provided greater support for the evolutionary relationships of phyllostomid monophyletic clades. This presents a unique opportunity to map primitive and derived chromosomal character states across different lineages in statistically supported trees [1,9-12].

Here, we made chromosome paints from flow-sorted chromosomes of *M. californicus* (MCA, diploid and fundamental numbers: 2n = 40; FN = 60), subfamily Macrotinae, to assist in documenting the pattern of karyotypic evolution during the phyllostomid radiation. We have chosen *M. californicus* for generating chromosome-specific painting probes based on the following reasons: 1) hypothetically it has a karyotype that differs by only three centric fusions from the ancestral karyotype of Phyllostomidae (*M. waterhousii*, MWA, 2n = 46; FN = 60) [2,13]; 2) it has a significant number of acrocentric chromosomes proposed as conserved chromosome arms in other phyllostomid species [13] and; 3) the higher diploid number can provide a larger number of probes, and thus allows detailed comparisons.

In the present study, we have chosen the nectar-feeding phyllostomids, a particularly notorious group in terms of dietary adaptations and phylogenetic position, to test the efficiency of MCA chromosomal probes as a tool for identifying homologous regions among species with highly rearranged karyotypes. Although historically all nectar feeders were considered a monophyletic group ([14] and references therein), recent gene trees with strong statistical support evidenced that the subfamily Glossophaginae diverged from the basal clade first, followed by an independent divergence from the basal stock that gave rise to Lonchophyllinae and four other subfamilies [9]. In addition to the molecular data, further evidence for convergent origin of nectar feeding is documented from morphological analyses [9,10,15,16]. Previous data on classical G-banding have suggested that chromosomal evolution within nectar-feeding phyllostomids might reflect the diphyletic origin for Lonchophyllinae and Glossophaginae [17]. However, the frequent occurrence of rearrangements other than Robertsonian (Rb) fissions and fusions within the main lineages of nectar-feeders has prevented an unambiguous identification of banding homology among species with distinct karyotypes [18].

Herein, we examined chromosomal homologies between two representatives of the Glossophaginae (*Glossophaga soricina* - GSO and *Anoura cultrata* - ACU) and one species from the Lonchophyllinae (*Lonchophylla concava* - LCO). Our goals were to find shared syntenic associations among the studied species, as well as to map chromosome characters for the different nectar feeding lineages and *M. californicus* on a DNA-based phyllostomid tree [9]. Our cross-species painting results were presented using a phylogenetic approach for chromosome changes, correlating cytogenetic and DNA sequence data.

## Methods

### Generation of *Macrotus californicus* (MCA) whole chromosome paints

Five specimens of *M. californicus* were collected at Picacho Peak Mine at the Picacho Peak State Park, Pinal County, AZ. 32.655°N42°W, USA. The specimens, including skeletal material, tissue samples, cell lines, and chromosomal preparations were deposited at the Natural Science Research Laboratory (NSRL) at the Museum of Texas Tech University under the identification codes TK163823 – TK163827. All specimens for this study were collected in accordance with animal welfare guidelines established by the Texas Tech University Animal Care and Use Committee.

Primary cell lines from ear and lung biopsies of all specimens were established at the Department of Biological Sciences, Texas Tech University. The chromosomes obtained from the cell line of a male specimen (TK165824) were sorted on a MoFlo dual-laser cell sorter and isolated according to size and base-pair composition [16] at the Wellcome Trust Sanger Institute, UK. The chromosome-specific probes were prepared and labeled by degenerate oligonucleotide primed-PCR (DOP-PCR) on individual flow-sorted chromosomes as previously described [19,20].

### *In situ* hybridizations

Cross-species chromosome painting was performed according to Yang et al. [20] and Volleth et al. [4] with slight modifications. Namely, generated probes were hybridized onto *in vivo* bone marrow karyotypic preparations of *G. soricina* (2n = 32; FN = 60; TK101019, male), *A. cultrata* (2n = 30; FN = 56; TK104201, male) and *L. concava* (2n = 28; FN = 50; TK104582, male). Due to the long storage time of the chromosome suspensions (approximately 12 years), long denaturation time was required for most of the slides (5–15 min, 70% formamide/30% 2XSSC (v/v) at 75°C). The detections were performed using Cy3 or FITC-Streptavidin (1:500, Amersham Biosciences) and Anti-Digoxigenin-Rhodamin, Fab fragments (5:1000, Roche Applied Science), and the chromosomes were counterstained with DAPI (4′,6-diamidino-2-phenylindole). No Y chromosome paint was used in our cross-species hybridizations.

### Data analysis

For the chromosomal identification of the flow-sorted peaks, the generated probes were hybridized back to MCA mitotic metaphases. Additionally, G-banding and DAPI-banding techniques were performed to establish a reference set of MCA chromosomes [21]. The banding pattern provided by the counterstain DAPI during FISH experiments in conjunction with the G-banding patterns for each species were used to assign the hybridization signals onto specific chromosomes of the studied taxa. Our data were compared to previous findings derived from chromosome painting and G-banding of GSO and other nectarivorous bats [4,18,22,23]. Additionally, MCA, ACU, and LCO chromosome segments were related back to human chromosomes (HSA), using the homologies proposed among GSO and HSA [23]. Finally, the shared syntenic associations revealed by comparative chromosome painting were analyzed in a phylogenetic context by observing their position relative to branches on the phylogenetic tree of Baker et al. [9] to better understand the changes that have shaped chromosomal evolution among nectarivorous bats. The images not depicted in the present manuscript, including additional G-banded karyotypes and *in situ* hybridizations with MCA specific probes, are available upon request.

## Results

### Characterization of MCA painting probes

**Figure 1:**
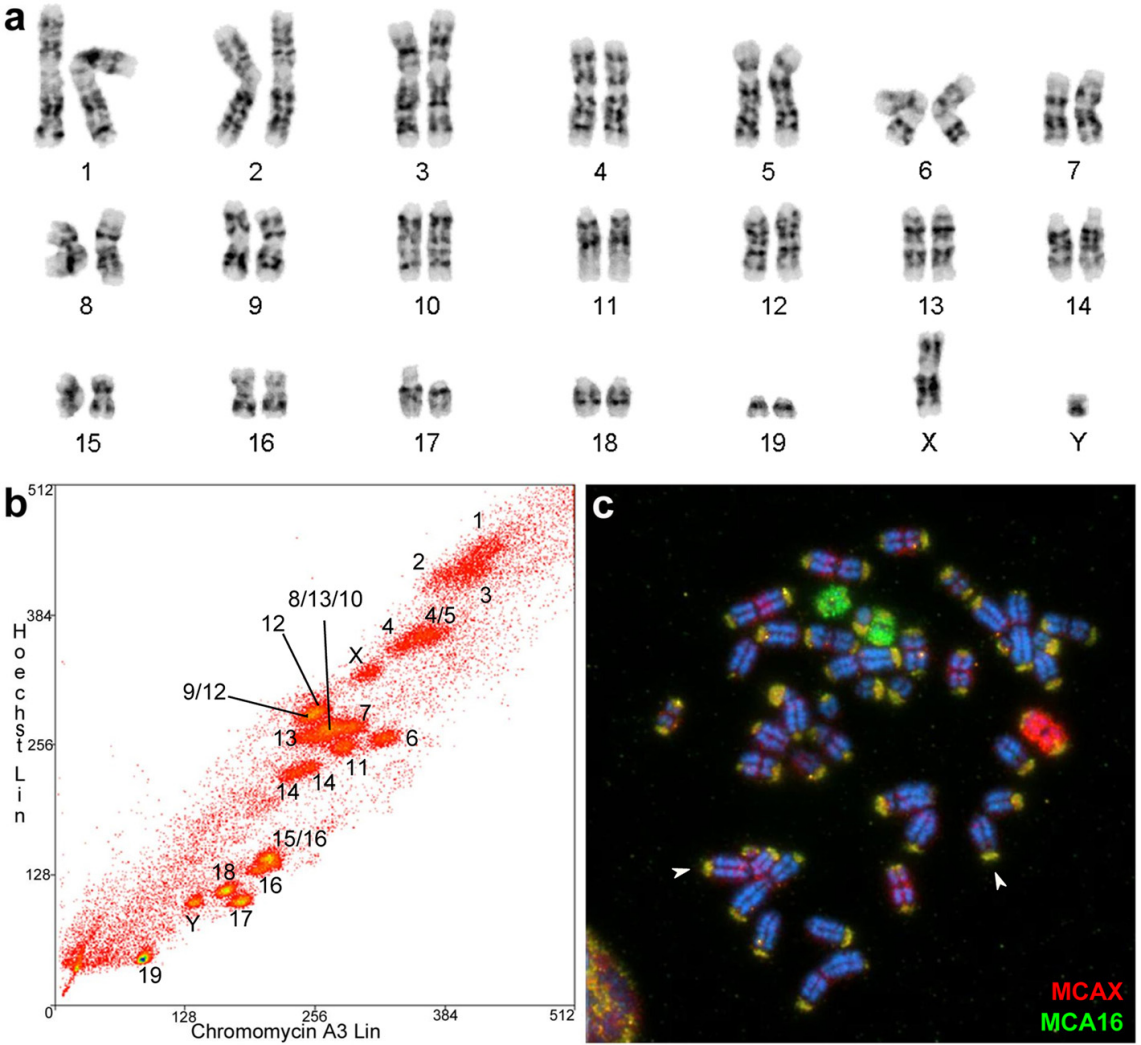
**Characterization of MCA whole chromosome paints: (a)***Macrotus californicus* G-banded karyotype; **(b)** flow cytometry peaks and corresponding chromosomal pairs; **(c)** FISH of the generated probes (X in red, 16 in green) on MCA karyotype. The arrows point to two examples of the telomeric repetitive DNA pattern revealed by the probes used. Note that this pattern is present in all chromosomes.

The 40 MCA chromosomes (standardized in Figure 1a) were resolved into 21 peaks (Figure 1b). Seventeen peaks each contained DNA from a single MCA chromosome, whereas four peaks each comprised more than one type of MCA chromosomes. For the latter, combined analysis of probes (e.g. comparison of the signal detected by chromosomes that were isolated in both, individual and shared peaks) allowed us to identify the corresponding MCA chromosome on the karyotypes of nectarivorous bats. Additionally, preliminary hybridization results have shown that MCA8 corresponds to chromosome 12 of the vespertilionid bat *Myotis myotis* (MMY). We have used MMY 12 probe [24], together with the isolated peak of MCA13 to differentiate MCA chromosomes 8, 10, and 13, which were present in the same flow sorting peak (Additional file 1: Figure S1). Some MCA chromosomes were sorted into different peaks due to differential repetitive DNA content among homologous. This was the case for MCA chromosomes 4, 14, 12, 13, and 16).

One particularity of MCA genome, evidenced by the hybridizations of the generated paints back to MCA karyotype, is the substantial amount of repetitive DNA located at the telomeric regions of MCA chromosomes (Figure 1c, Additional file 1: Figure S1). In experiments with lower stringency or without the use of blocking DNA, such as Cot-1, all MCA chromosome ends were labeled, regardless of the chromosomal probe used. This pattern was not recovered in hybridizations with MCA probes onto the chromosomes of the nectarivorous bats examined.

### Hybridization of MCA painting probes onto metaphase chromosomes of nectar-feeders

**Figure 2:**
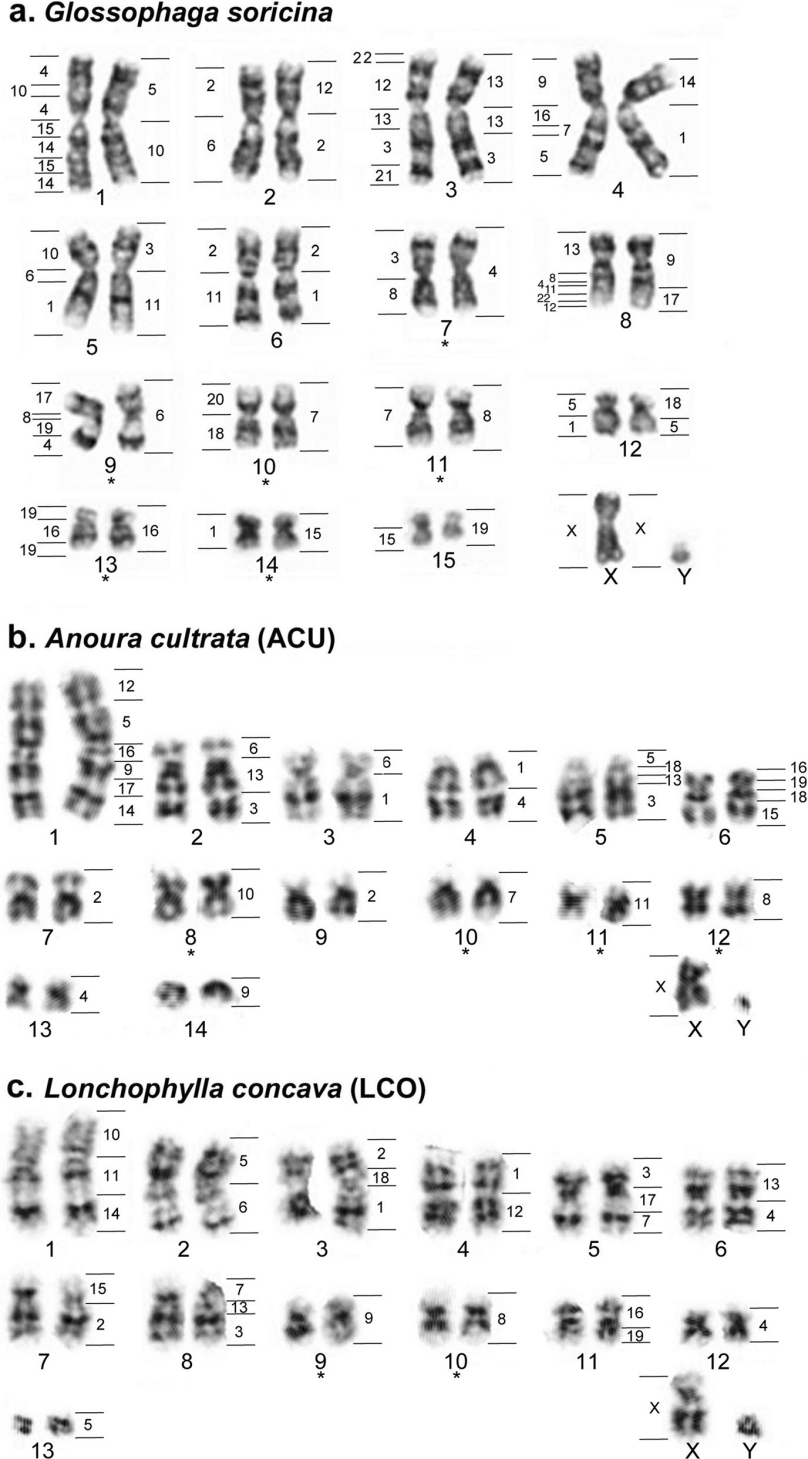
**MCA homologous chromosomal regions mapped on G-banded karyotypes of nectarivorous phyllostomid bats: (a)** GSO; **(b)** ACU; **(c)** LCO. * corresponds to conserved MCA chromosomes. In addition, homology to human chromosomes is indicated to the left of each GSO pair (data from [23] and unpublished results). The following segments were detected recently: GSO 3qp: HSA 13; GSO 8qi: HSA 4; GSO 9qp: HSA 8; GSO 13qt: HSA 19. The Y chromosome of ACU is derived from a different metaphase spread, and had its relative size corrected for the image. G-banding images of GSO modified from Volleth et al. [23], Figure 1a, p. 59, with kind permission of Springer Science and Business Media.

**Figure 3:**
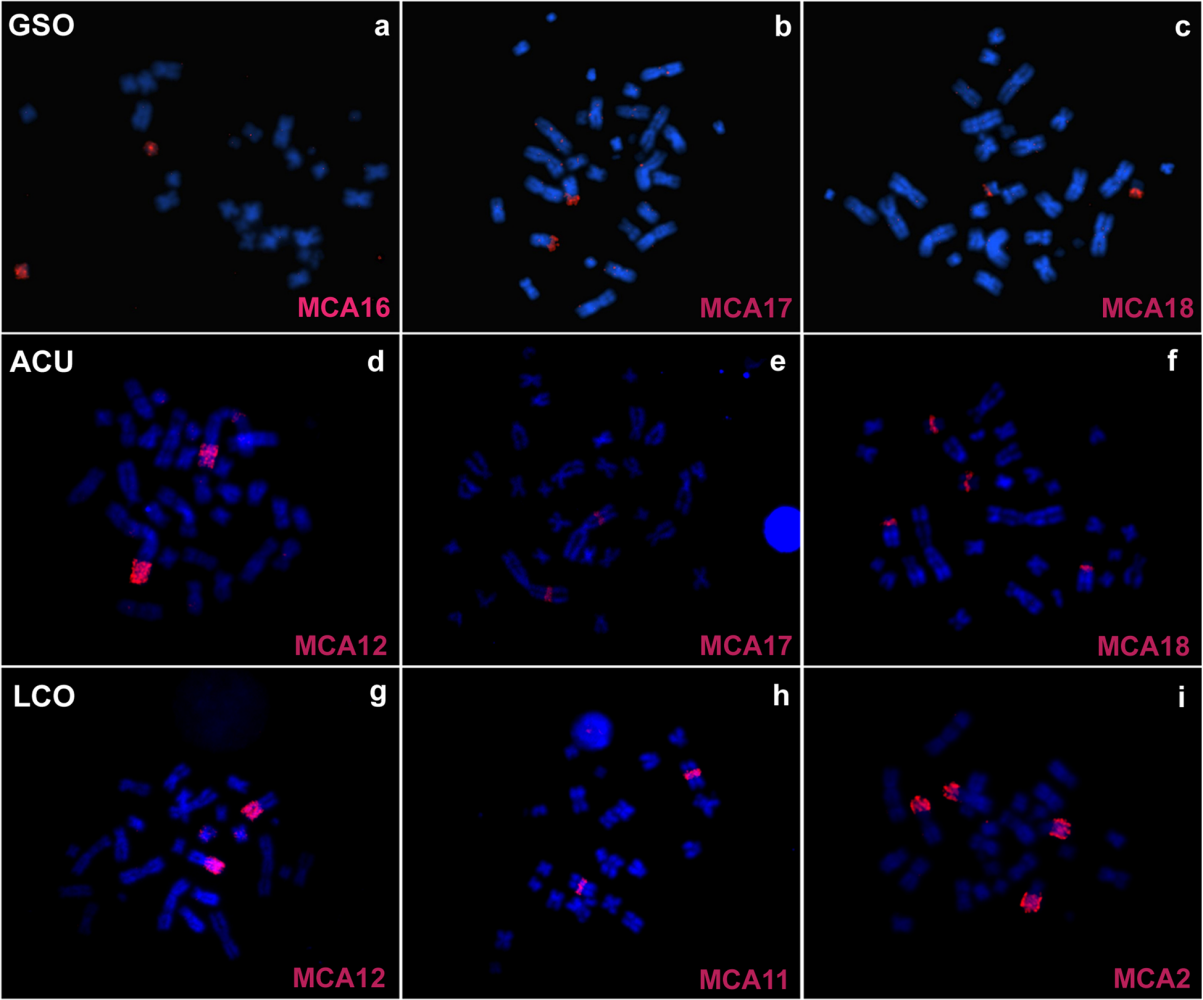
**Representative images of in situ hybridizations with *Macrotus californicus *(MCA) chromosome paints on nectarivorous metaphases.** The painting probes used in GSO **(a-c)**, ACU **(d-f)**, and LCO **(g-i)** metaphases are indicated in pink in the lower corner of each picture.

The hybridization results of MCA paints onto metaphase chromosomes of the three studied species are summarized in Figure 2, whereas examples of *in situ* hybridization images using MCA probes on the karyotypes of the nectarivorous are shown in Figure 3. Each of the probes used has detected at least one homologous chromosomal region in the karyotypes analyzed. The paint probes representing the 19 autosomes of MCA detected 24, 29, and 26 conserved chromosomal regions in GSO, ACU, and LCO, respectively. As expected, the X chromosome appears as a single conserved block in all four species.

Overall, the GSO karyotype was the most conserved among the three species studied, displaying six intact MCA autosomal pairs (MCA 4, 6, 7, 8, 15, and 16) and one conserved block on the NOR-bearing smallest pair (bi-armed GSO 15 but acrocentric MCA 19). Most of GSO derived chromosomes are combinations of two different MCA chromosomal arms through the centromere, except GSO 3, 8, and 12, which may be derived from terminal chromosomal fusions (Figure 2a). In contrast, the karyotypes of ACU and LCO were highly derived. ACU presented complete synteny of only four MCA chromosomes (MCA 7, 8, 10, and 11), with a total of four ACU chromosomes each corresponding to more than two MCA chromosomes (ACU 1, 2, 5, and 6 – Figure 2b); the remaining ACU chromosomes were each homologous to two MCA chromosomes or chromosomal segments. The combined analysis of the whole chromosome paints hybridizations with the assessment of chromosome morphology and banding patterns indicates that three of the four MCA conserved chromosomes have undergone inversion(s) or centromeric shifts in ACU’s karyotype. Namely, MCA 7 is a submetacentric element in contrast with the subtelocentric homolog ACU 10, and the acrocentric MCA 10 and 11 appear as metacentric chromosomes in the homologous ACU 8 and 11, respectively. LCO shares only two intact autosome pairs with MCA (MCA 8 and 9) and presented four chromosome pairs each homologous to more than two MCA chromosomes (LCO 1, 3, 5, and 8 – Figure 2c).

**Figure 4:**
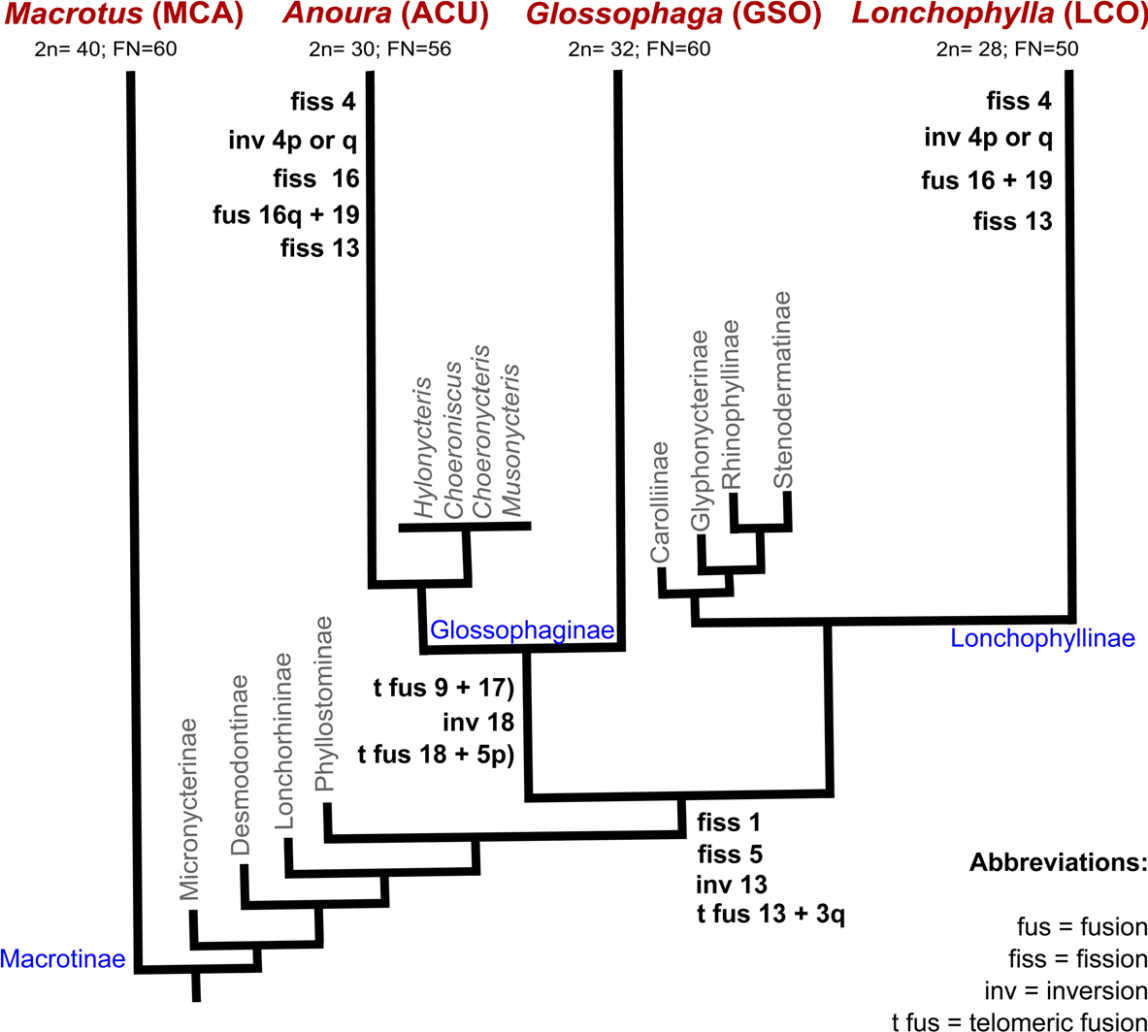
**Mapping of shared chromosomal rearrangements among the nectar feeders on the molecular phylogeny of Phyllostomidae.** The rearrangements were inferred using MCA karyotype as outgroup and are depicted in terms of MCA chromosome numbers. When a rearrangement was present in all three nectar-feeding species analyzed, it was plotted at the node corresponding to their most recent common ancestor. The rearrangements shared by ACU and GSO were plotted on the common ancestor of all glossophagines, whereas the rearrangements shared between ACU and LCO not found in GSO were mapped at terminal nodes. GSO presents MCA 4, 16, and 19 as individual chromosomes (similar condition as in MCA). In the case of chromosomes 13, 17 and 18, an acrocentric condition as in MCA was assumed for the last common ancestor of the nectar feeders to deduce the chromosomal rearrangements. The phylogenetic relationships are based on the previous published tree of Baker et al. [9]. For simplicity, we have omitted the sister genera for GSO and LCO.

A single MCA chromosome (MCA 8) appears conserved in all three lineages, whereas an additional entire MCA autosome was shared between GSO and ACU (MCA 7) as a single block but with different morphology/banding patterns (metacentric in GSO and subtelocentric in ACU). Our analysis revealed a single syntenic association shared among the three studied species (MCA 13/3), two potential chromosome signatures uniting ACU and GSO (MCA 9/17 and MCA 18/5) and one syntenic association shared between LCO and ACU (MCA 16/19). The mapping of these syntenic associations on the phylogenetic tree of phyllostomid bats [9], together with proposed shared rearrangement between the three nectarivorous bats studied, is shown in Figure 4. The karyotypic comparison among the studied species and human (HSA) chromosome segments in light of the Evolutionarily Conserved Units (ECUs) proposed by Volleth et al. [4] is summarized in Table 1, where we propose the correspondence of ECUs to chromosome segments of the phyllostomid species studied herein.

**Table 1:** **Chromosome homologies between *M. californicus *** **(MCA), *A. cultrata *** **(ACU), *L. concava *** **(LCO), *G. soricina *** **(GSO), and chiropteran ECUs**

**MCA**	**ACU**	**LCO**	**GSO**	**ECUs**
1p	4qp	4p	6q	11a
1q	3q	3q	4q	5a:7b:16b*
2p	7 or 9	3 pt or 7q	6p	2b
2q	7 or 9	7q or 3pd	2q	6a
3p	5q or 2qd	5p	5p	10a
3q	2 qt or 5q	8q	3qd	3a-21^
4p	4qd or 13	6q or 12	7p	3b
4q	4qd or 13	6q or 12	7q	8a
5p	5p	13	12 qt	1c
5q	1 pp	2p	1p	4a:10b*
6p	3p or 2 pt	2qp	9p	17
6q	2 pt or 3p	2 qt	9q	4b:8:19b* (4-8^)
7	10	8p + 5 qt	10	18:20*
8	12	10	11	7a
9	1qi + 14	9 Inv	8p/qp	13a:8b-4c*#
10	8 bi-armed	1p	1q	14a-15a-14b-15b (14-15^)
11	11 bi-armed	1qp	5q	1a-6b *
12	1 pt	4q	2p	2a
13	2qp + 5qi	6p + 8qi	3p/qp	12a-22a^+13b #
14	1 qt	1 qt	4p	9
15	6qd	7p	14	1b
16	1qp + 6 pt	11p/qp	13	16a:19a^
17	1qi	5qp	8 qt	11b-22b-12b*
18	5qp + 6qp	3 pp	12p/qp	5b
19	6 pp	11 qt	15	15c

Evolutionarily conserved units (ECUs) proposed by Volleth et al. [4] as human chromosomes syntenic associations building chiropteran karyotypes (last column). Slash = connection with centromere (/); DASH = connection without centromere (-); Colon = Both possibilities have been found (:). * = Chiroptera specific; ^ = mammalian; a, b, c indicate different segments, “a” being the largest. #=A small HSA13 homologous segment, i.e. 13b, was separated from ECU13:8b-4c, resulting in 13a:8b-4c. Such arrangement has been frequently found in Vespertilioniformes. p: short arm of a bi-armed chromosome; q: long arm; p: proximal part; d distal part; t: terminal part; i: interstitial segment.

## Discussion

### FISH with *Macrotus californicus* chromosome paints

In this study, *M. californicus* (MCA) chromosomal probes were used to determine karyotypic homologies among three nectar-feeding phyllostomid bats. The unusual pattern of cross-hybridization to centromeric and telomeric regions observed in the hybridizations of MCA probes on MCA chromosomes did not interfere with the subsequent cross-species chromosome painting. This, together with the finding that hybridizations with telomeric (TTAGGG) sequences as probes revealed telomere-only distribution patterns on the chromosomes of *M. californicus* and *M. waterhousii*, indicates that besides the conserved telomeric sequences, the chromosome termini of MCA are comprised of large amount of repetitive DNA, which might be unique to this species [25]. The rapid divergence of repetitive sequences within phyllostomids is probably the main contributing factor to the success of MCA probes in cross-species painting [26].

Overall, our chromosome painting data show extensive chromosomal reorganization among MCA and nectarivorous phyllostomids. Using MCA as the outgroup for comparing the three nectarivorous species, we have been able to infer that the GSO karyotype has a higher number of shared intact chromosomes with MCA. Baker and Bass [22] G-band analysis resulted in the identification of five chromosomal pairs conserved between GSO and *M. waterhousii*, which correspond to five out of the six MCA/GSO conserved chromosomes detected in this study (MWA 4/5 = GSO 7 = MCA 4; MWA 6/7 = GSO 9 = MCA 6; MWA 15/16 = GSO 10 = MCA 7; MWA 19/20 = GSO 11 = MCA 8; and MWA 25/26 = GSO 14 = MCA 15). Applying parsimony to our data, and given the presence of the same intact chromosome blocks in such distantly related species, we propose that these chromosomes plus GSO 13 (= MCA 16) were present in the common ancestor of phyllostomid bats, as well as at the base of the clade comprising Glossophaginae, Lonchophyllinae, and the other four subfamilies that form a monophyletic group sister to the lonchophyllines (i.e. Carolliinae, Glyphonycterinae, Rhinophyllinae, and Stenodermatinae). The presence of this high number of conserved chromosomes at the base of each subfamilial lineage suggests a conserved chromosomal landscape before the radiation of ecologically unique monophyletic subfamilies. Increased rearrangement activity, however, is observed in specific clades within each subfamily.

As an example of unique genomic reorganization, the clade comprising *Anoura* underwent fixation of distinct chromosomal rearrangements after splitting from the glossophagine common ancestor. Although ACU shares four chromosomes with MCA, the occurrence of substantial genomic reorganization can be exemplified by its largest autosomal pair. The high variety of syntenic associations within this chromosome suggests that rearrangements such as inversions and tandem fusions, which are expected to be highly deleterious to the production of balanced gametes in meiosis, have achieved fixation during the evolutionary history of this lineage. Additionally, disruption of MCA chromosomes not observed for the other studied species are common, such as MCA 9, 16, and 18, that might have originated by Rb fissions or whole arm translocations with subsequent fusions to form the extant chromosome blocks (Figures 2 and 4). Interestingly, banding comparison suggests that although the karyotypes of all *Anoura* species are indistinguishable, karyotypic variation is observed among genera within its sister clade, which comprises *Hylonycteris* (2n = 16; FN = 24), *Choeroniscus* (2n = 19–20; FN = 32–36), *Choeronycteris* (2n = 16; FN = 24–26), and *Musonycteris* (2n = 16; FN = 22) [17,18,27]. Further cross-species chromosome painting analyses among species in this clade would provide insights into the extent of variation within this complex lineage.

The karyotype evolution of LCO has generated multiple chromosomal character states, most of which appear to be autapomorphic, and have not been found in the karyotypes of MCA or glossophagines examined thus far. The mapping of chromosome rearrangements on the molecular phylogenetic tree of Phyllostomidae has revealed that some rearrangements in LCO have also occurred in ACU but not in GSO karyotypic evolution (Figure 4). The chromosomes involved in these shared rearrangements in ACU and LCO are present as individual chromosomes or single syntenic blocks in GSO and MCA, which indicates that convergent rearrangements might also have played a role in shaping the karyotypes of extant phyllostomid bats.

The high number of chromosome rearrangements leading to autapomorphic syntenic associations present in ACU and LCO karyotypes presents an interesting landscape to discuss the fixation of multiple chromosome rearrangements in specific mammalian lineages. Historically, most of the models aiming to explain how chromosome changes get established in natural populations were derived from theoretical work, and it has been difficult to test if the proposed scenarios occur in nature (see [28] for a review). Throughout the years, the model of fixation of chromosome rearrangements due to drift in small, inbreeding populations has been used to explain the establishment of rearrangements accepted as conferring disadvantages and reducing fertility in heterozygous [29]. The weakness of this model to predict the frequency of rearrangements in natural populations is discussed in [30-32], and empirical datasets demonstrating the role of bottlenecks, small deme size, and inbreeding as drivers of fixation of new chromosome forms are scarce in the literature.

Recently, there has been a greater acceptance of the suppression of recombination model of speciation [33,34]. This model postulates that chromosome rearrangements can become fixed and serve as isolating barriers by the accumulation of selectively advantageous mutations in chromosomal regions protected from recombination by a given rearrangement. This model circumvents the flaws in the negative effects on meiosis in heterozygous for a given rearrangement and presents the advantage of being testable in natural populations. There is growing evidence from molecular data of positive selection of genes within rearranged areas (e.g. human, *Drosophila*, *Anopheles*), and actual examples of distinct phenotypic traits between populations with different chromosomal forms in nature [35,36]. Differential phenotypes due to suppression of recombination and selection for specific alleles within rearranged areas might be a parsimonious way to achieve karyotypes with multiple fixed rearrangements that in other circumstances would be highly deleterious in the heterozygous state and improbable to achieve fixation even in populations subject to multiple bottlenecks. Thus, the unique rearrangements presented by the nectar feeders analyzed herein, especially the multiple autapomorphic syntenic associations of ACU and LCO, remain candidates for adaptive rearrangements that facilitated directional selection for a new niche. This is a theoretical position and, like the drift in small populations model, must be tested by empirical methods.

### Evolutionarily conserved units (ECUs) in Phyllostomidae

According to Volleth et al. [4] the chiropteran karyotypes can be reconstructed from 25 conserved chromosomal blocks referred to as Evolutionarily Conserved Units (ECUs). Our hybridization results for MCA probes onto GSO chromosomes allowed us to try to relate the karyotypes of the MCA, ACU, and LCO species back to human (HSA) chromosome segments. For this, previous chromosome homologies among GSO and HSA from Volleth et al. [23] were integrated with data derived from MCA paints on GSO karyotype. The last column of Table 1 shows these ECUs as HSA chromosomal segments and their correspondence in MCA and nectar-feeders. All ECUs identified by Volleth et al. [4] are present as entire chromosomes or chromosome arms in MCA and GSO, which suggests that their karyotypes were formed mainly by reshuffling of ECUs and not synteny disruption. The karyotypes of ACU and LCO, on the other hand, have shown synteny disruption for 4 and 2 ECUs, respectively, which is in agreement with the intense karyotypic evolution displayed by the two genera.

Although tentative, the homology assignment among the ECUs in MCA and nectar feeders through the integration of the painting results with the G-bands has revealed conserved chromosome blocks for at least five ECUs. Given the limited resolution of the nectar-feeder’s G-bands due to the chromosome condensation of the bone marrow preparations, our comparison was made mainly by assessing the relative position of the darker G-bands within each segment. Taking that into consideration, we propose these syntenies (HSA 2a, 5a:7b:16b, 7a, 9, and 11a) were present in the most recent common ancestor of all phyllostomid bats. Other ECUs seem to be conserved (e.g. HSA 2b, 3b, 8a, 10a, and 12a-22a), but further evaluation is needed. Overall, the integration of the published GSO/HSA chromosome homology map with the correspondent homologies among MCA, GSO, ACU, and LCO only allows a glimpse of ECUs conservatism in the phyllostomids analyzed (Table 1).

Interestingly, we observed two syntenic associations that are potential chromosome synapomorphies for the subfamily Glossophaginae (MCA 18/5 and 9/17, corresponding to HSA 5b/1 and 13-8-4:11b-22b-12b, respectively). Both HSA chromosomes 5 and 1 are proposed to have been present in the ancestral eutherian karyotype (AEK) as single syntenic blocks [7,37-39]. Murphy et al. [40] have shown multiple breakpoints within the ancestral chromosome 1 among different mammalian orders, whereas HSA 5 has been shown to be conserved in xenarthrans but was involved in different rearrangements across distant mammalian lineages. HSA 1/5 syntenic associations have been reported as synapomorphic conditions within a small number of monophyletic taxa: it has been shown as a link between the Eulipotyphla families Erinaceidae and Talpidae [41,42], and in Artiodactyla there are two syntenies including HSA 1 and 5 in pig and cow (HSA 1q/5q/19p and 1q/5pq) [42]. Contrastingly, an HSA 5q/1a/19q association has been reported as an autapomorphy for the species *Galago moholi* within the Lorisiformes [43]. In bats, the association 5b/1c found in *Glossophaga* has been proposed to be a characteristic feature within Phyllostomidae [4]. The finding that this association is not found in other bats, including *M. californicus*, might be indicative of a synapomorphy uniting the Glossophaginae (see Figure 4). Alternatively, if *M. waterhousii’s* chromosome arm 14 is homologous to GSO 12, as proposed by Volleth et al. [4], this association would mark a chromosome signature for the family, with disruption of this linkage group in MCA appearing after its divergence from MWA. Further studies with other phyllostomids as well as phyllostomid sister groups are required to reject either of these alternative hypotheses.

We have identified a second potential synapomorphy for the subfamily Glossophaginae, MCA 9/17, which is the putative association of HSA 4c:11b from the ECUs 13-8b-4c and 11b-22b-12b. In GSO, the synteny seems to be formed by a fusion of the acrocentric element MCA 17 to the terminal portion of the short arm of the MCA 9. However, although the association of MCA 9/17 in ACU 1q seems to correspond to GSO 8q, it could also have been formed independently among the two species, or constitute a plesiomorphic character with a derived state in MCA. Therefore, a definitive assessment of this syntenic association will only be possible after chromosome painting with HSA probes is performed on MCA and ACU karyotype, and after further comparative analysis with other phyllostomids and outgroups.

### The application of MCA whole chromosome paint probes to Phyllostomidae chromosome evolution studies

A recent finding with major implications from a cytogenetic standpoint is the proposed position of the two species of *Macrotus* (in a new subfamily, Macrotinae) as the basal clade for the family Phyllostomidae in all trees derived from molecular data [1,9-12]. Interestingly, previous cladistic cytogenetic studies using G-bands as characters propose the karyotype of *Macrotus waterhousii* (MWA, 2n = 46; FN = 60) as the ancestral for the family [13]. Although the hypothesis of retention of primitive character states by *Macrotus* can only be tested when proper comparative chromosome painting analysis is performed between *Macrotus* and phyllostomid outgroups, we propose plesiomorphic chromosomes for the family, based on their presence in MCA’, and at least one of the nectar feeders studied. The differences involving these syntenic blocks among nectarivorous studied herein are presented on Table 1.

The karyotypic variability of the nectar-feeding phyllostomids presented herein is a further example of chromosome painting as a powerful technique for the identification of homologous chromosome regions, in comparison to classical banding [19,20]. G-band analyses of nectar-feeding species with identical karyotypes, such as the glossophagine clade comprising *Glossophaga*, *Monophyllus*, *Phyllonycteris*, *Erophylla*, and *Brachyphylla*, have been accurate enough to support the grouping of these five monophyletic lineages despite of their considerable morphological variation [22]. In contrast, similar analyses of nectar feeders with highly rearranged karyotypes, such as the identification of chromosomal homologies among *Anoura caudifer* and *Glossophaga soricina*), resulted in no more than two recognizable syntenic associations. This lack of resolution of G-banding in cases of substantial chromosomal reorganization, such as the nectar feeders studied herein, makes it inadequate for the use of chromosome information as phylogenetic characters to understand classification [18].

Although G-banding data have provided insights into the paraphyly of nectarivorous phyllostomids [17,18], the identification of shared syntenic associations through chromosome painting, which can be used as markers for a cladistic analysis, will be of fundamental importance in linking chromosomal data with phylogenies generated by different datasets. Considering each identified syntenic association as a character, our results would provide at least nine characters that could be used in cladistic studies of phyllostomid evolution. Pieczarka et al. [8] and Sotero-Caio et al. [44] provide the first attempts to use chromosome painting syntenies in a cladistic framework to test intergeneric relationships within Phyllostomidae. The integration of data from MCA chromosome paints with those from *Carollia brevicauda* and *Phyllostomus hastatus*[5] promises to provide enough information to generate a complete classification of Phyllostomidae through chromosome data in the near future.

Rapid diversification rates and extreme morphological and ecological variability in phyllostomids are coupled with high rates of chromosomal change, which makes them an ideal model system to study the roles of chromosomal rearrangements in speciation and morphological/ecological variation [2,12,45]. Chromosomal change information in the mammalian family with the widest range of adaptations for distinct dietary strategies is key to understanding karyotypic evolution processes and the role of chromosomes in diversification. The use of MCA probes to define regions of chromosomal homology among species will significantly contribute to genomic resources for the bat research community, as well as studies on evolution of chromosomes and their contribution to diversity and adaptation of mammals.

## Conclusions

Our data have shown that a large number of chromosome rearrangements is responsible for the observed variation among the karyotypes of *Glossophaga soricina*, *Anoura cultrata* (Glossophaginae) and *Lonchophylla concava* (Lonchophyllinae). The syntenic association analysis suggests that the karyotypic evolution of nectar-feeders was marked not only by reshuffling of chiropteran Evolutionarily Conserved Units (ECUs) through Robertsonian rearrangements, but also by less common rearrangements that might play a key role in genome reorganization within the family. Therefore, further application of *M. californicus* chromosome paints in comparative cytogenetics of Phyllostomidae will help the understanding of chromosomal evolution patterns among phyllostomid bats and will help elucidate the role of karyotypic change as a possible source of phenotypic variation within the family.

## Abbreviations

2n: Diploid number; 2XSSC: Saline-sodium citrate buffer; ACU: *Anoura cultrata*; DAPI: 4′,6-diamidino-2-phenylindole; DOP-PCR: Degenerate oligonucleotide primed polymerase chain reaction; EAK: Eutherian ancestral karyotype; ECU: Evolutionarily Conserved Unit; FN: Fundamental number of autosomal arms; GSO: *Glossophaga soricina*; HSA: *Homo sapiens*; MCA: *Macrotus californicus*; MMY: *Myotis myotis*; MWA: *Macrotus waterhousii*.

## Competing interests

The authors declare that they have no competing interests.

## Authors’ contributions

CGSC conducted the field work to obtain the MCA biopsies, carried out the establishment of cell lines, FISH experiments, performed the analysis and interpretation of data, and drafted the manuscript. MV helped with the FISH experiments, analysis and interpretation of data, and drafting of manuscript. LSG supervised the establishment of MCA cell lines, helped with the tissue culture work and with drafting of manuscript. BF performed tissue culture work and FISH on MCA karyotype. WC carried out the acquisition of the flow sorting data. BLN supervised the flow sorting work, helping with critical optimization of sorting protocols. FY participated in the design of the study, acquisition and interpretation of data and drafting of manuscript. RJB conceived the study, participated in its design and coordination, conducted the field work to collect all samples, helped in the interpretation of data and with drafting of manuscript. All authors read and approved the manuscript.

## Supplementary Material

Additional file 1: Figure S1 Identification of *Macrotus californicus* (MCA) chromosomes 7–14 using multicolor FISH on MCA karyotype **(a)**, and identification of MCA 8 on chromosomes of nectar-feeding phyllostomids using *Myotis myotis* (MMY) chromosome 12 probe **(b-d)**. In **(a)**, the multicolor FISH of MCA flow sorted peaks shows two examples of probes comprising more than one MCA chromosome pair (MCA 8/10/13 and MCA 9/12 in blue and in yellow, respectively). To distinguish between these chromosomes on the three nectarivorous bats analyzed, we used MCA chromosome-specific probes corresponding to MCA 13 and 12 (data not shown), together with MMY 12 = MCA 8. Examples of hybridizations using MMY 12 as a probe are shown for *Glossophaga soricina***(b)**, *Anoura cultrata***(c)**, and *Lonchophylla concava***(d)**. The white signals on the telomeres of MCA chromosomes in **(a)** derive from the cross-hybridization of shared repeats among different chromosomes, a unique characteristic of MCA genome. Click here for file
