# Supplementary figures and images for: Chromosomal evolution among leaf-nosed nectarivorous bats – evidence from cross-species chromosome painting (Phyllostomidae, Chiroptera)
Source: BMC Evol Biol. 2013 Dec 26;13:276. doi: 10.1186/1471-2148-13-276 (PMC3880000; doi:10.1186/1471-2148-13-276)

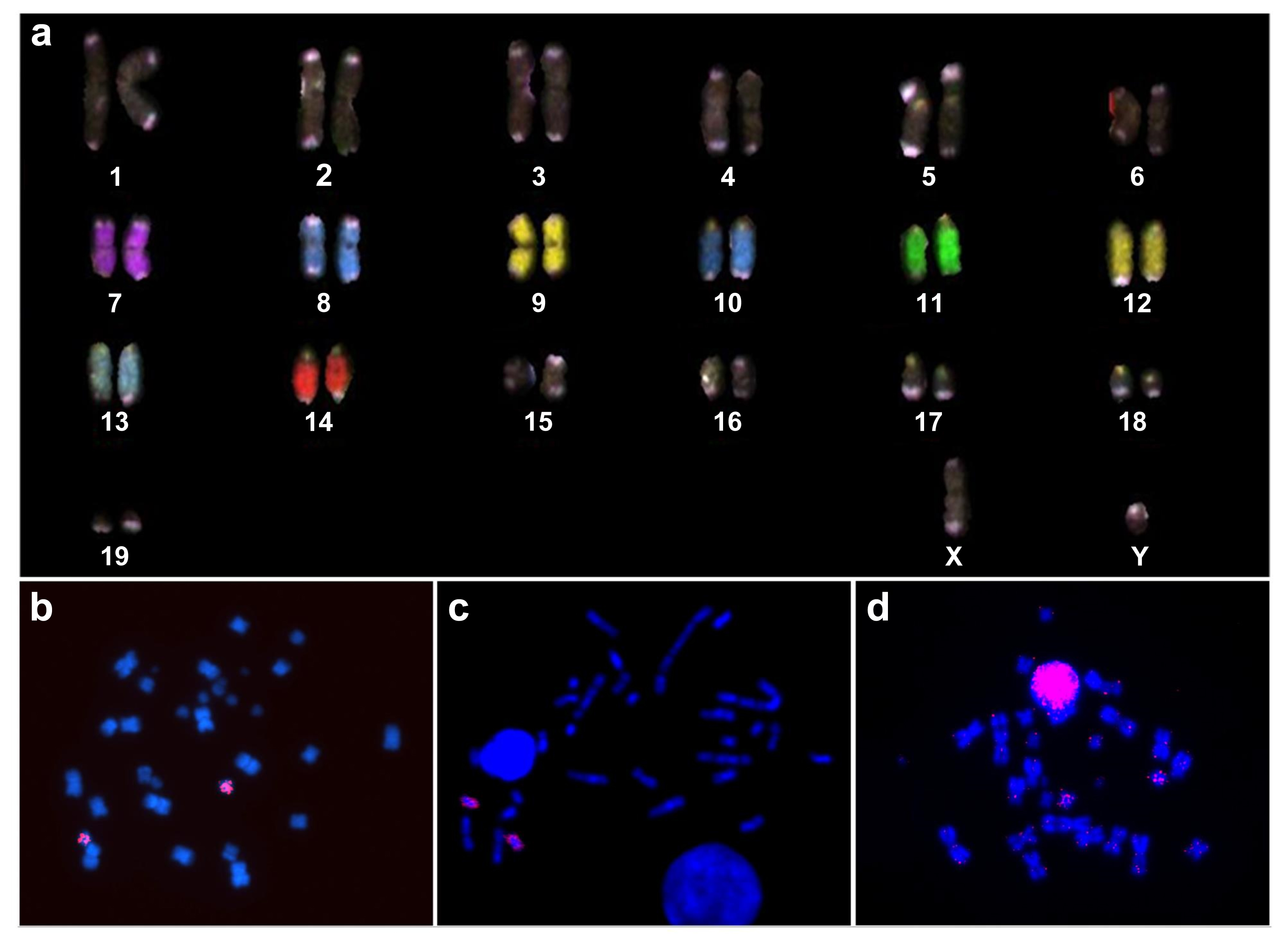

Supplement: Additional file 1: Figure S1 — Identification of Macrotus californicus (MCA) chromosomes 7–14 using multicolor FISH on MCA karyotype (a), and identification of MCA 8 on chromosomes of nectar-feeding phyllostomids using Myotis myotis (MMY) chromosome 12 probe (b-d). In (a), the multicolor FISH of MCA flow sorted peaks shows two examples of probes comprising more than one MCA chromosome pair (MCA 8/10/13 and MCA 9/12 in blue and in yellow, respectively). To distinguish between these chromosomes on the three nectarivorous bats analyzed, we used MCA chromosome-specific probes corresponding to MCA 13 and 12 (data not shown), together with MMY 12 = MCA 8. Examples of hybridizations using MMY 12 as a probe are shown for Glossophaga soricina(b), Anoura cultrata(c), and Lonchophylla concava(d). The white signals on the telomeres of MCA chromosomes in (a) derive from the cross-hybridization of shared repeats among different chromosomes, a unique characteristic of MCA genome. [file 1471-2148-13-276-S1.jpeg]
